# Intussusception in an Infant With SARS-CoV-2 Infection: A Case Report and a Review of the Literature

**DOI:** 10.3389/fped.2021.693348

**Published:** 2021-08-04

**Authors:** Massimo Luca Castellazzi, Antonio Corsello, Lucia Cerrato, Alessandra Carnevali, Anna Morandi, Ernesto Leva, Carlo Virginio Agostoni, Paola Marchisio

**Affiliations:** ^1^Fondazione IRCCS Ca' Granda Ospedale Maggiore Policlinico, Pediatric Emergency Department, Milan, Italy; ^2^University of Milan, Milan, Italy; ^3^Fondazione IRCCS Ca' Granda Ospedale Maggiore Policlinico, Radiology Unit - Pediatric Division, Milan, Italy; ^4^Fondazione IRCCS Ca' Granda Ospedale Maggiore Policlinico, Pediatric Surgery, Milan, Italy; ^5^Fondazione IRCCS Ca' Granda Ospedale Maggiore Policlinico, Pediatric Intermediate Care Unit, Milan, Italy; ^6^Department of Clinical Sciences and Community Health, University of Milan, Milan, Italy; ^7^Fondazione IRCCS Ca' Granda Ospedale Maggiore Policlinico, Pediatric Highly Intensive Care Unit, Milan, Italy; ^8^Department of Pathophysiology and Transplantation, University of Milan, Milan, Italy

**Keywords:** intussusception, SARS-CoV-2, COVID-19, gastrointestinal symptoms, infants

## Abstract

Coronavirus disease 2019 (COVID-19) is caused by acute respiratory syndrome coronavirus 2 (SARS-CoV-2). Even if predominantly considered a respiratory pathogen, it could be associated with gastrointestinal involvement, generally in mild forms. Recent reports highlight the association between SARS-CoV-2 and intussusception in infants. A case of intussusception is hereby described in a previously healthy infant in whom the diagnosis of SARS-CoV-2 was made after the analysis of bronchoalveolar lavage and intraoperative specimens following surgical procedures. Accordingly, a review of infant cases with intussusception and SARS-CoV-2 infection is also reported.

## Introduction

Intussusception, defined as the invagination of a proximal segment of the intestine into an adjacent distal segment, is considered a common cause of intestinal obstruction in patients with acute abdominal pain, especially in infants within 10 months of age. The most frequent form in pediatric age is the ileocolic (80%), followed by rare ileoileal and colocolic forms (1). Viral infections have been described as associated with this condition (2).

The severe acute respiratory syndrome-coronavirus-2 (SARS-CoV-2) was firstly identified in patients with a severe form of pneumonia in Wuhan in December 2019 and subsequently became pandemic (3).

The coronavirus disease 2019 (COVID-19) has been associated with a wide range of clinical presentations, from asymptomatic or mild respiratory symptoms to severe lung injury, multi-organ failure and death. However, it is now evident that SARS-CoV-2 can also affect the gastrointestinal system, with the highest incidence in pediatric age (4). Sporadic cases of intussusception in SARS-CoV-2 infected children have been reported worldwide (2).

Here, we report a case of a female infant with ileocolic invagination whose diagnosis of SARS-CoV-2 infection was achieved after an extended diagnostic workout. Furthermore, a literature review on this topic is also performed considering clinical presentation, diagnosis, treatment options and outcome.

## Case Presentation

A previously healthy 10-month-old girl was admitted to our emergency department for abdominal pain, accompanied by irritability, decreased oral intake, intermittent crying and two episodes of non-biliary vomiting during the previous 24 h. No fever or presence of bloody stools were referred in previous days.

On physical examination, she presented apyretic with good general conditions and normal vital signs. An abdominal distension was clearly observable, with crying and pain evoked by the palpation of the left lower quadrant. No signs of dehydration or respiratory distress were noted. A rectal probe was applied, which showed the presence of blood traces. Laboratory tests performed on admission showed a normal white blood cell count and a mild increase of C-reactive protein of 10.2 mg/L (normal value <5 mg/L). Renal, liver function tests and electrolytes resulted normal as well. Blood gas analysis was normal. A coproculture and research for adenovirus and rotavirus on stool sample were performed and subsequently resulted negative. Urine analysis did not show signs of urinary tract infection.

**Figure 1:**
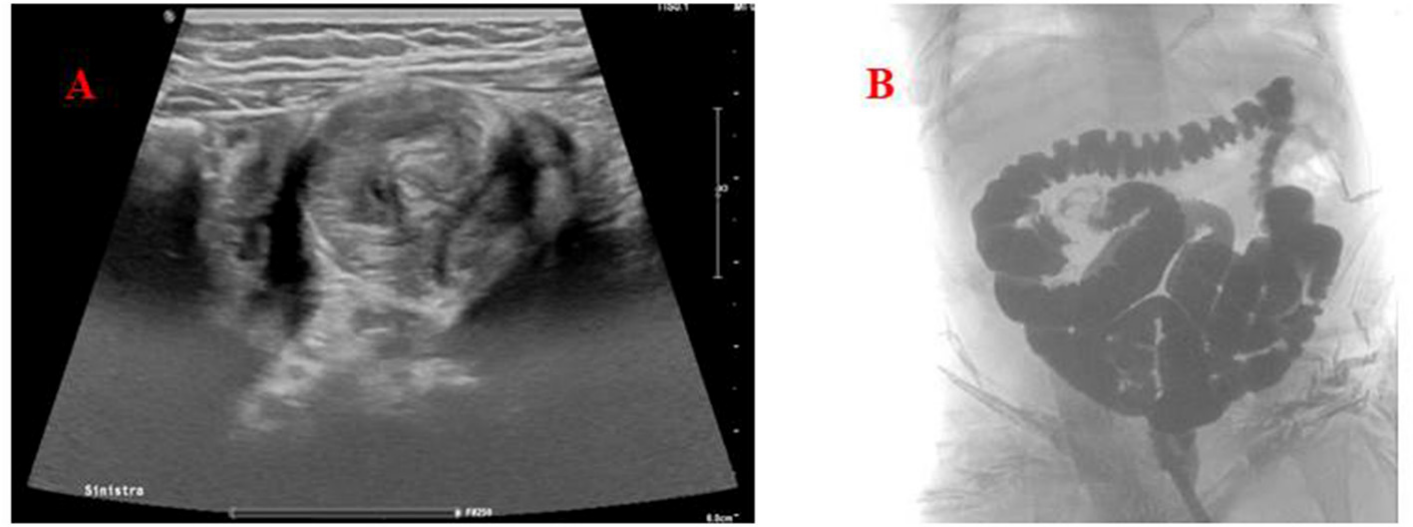
**(A)** Abdomen ultrasound. The target (donut) sign found in the left lower quadrant, confirming the diagnosis of intussusception. **(B)** Hydrostatic reduction of intussusception using Gastrografin® contrast solution showing the successful reduction of the ileocolic invagination.

Due to patient's age and clinical presentation, an abdominal ultrasound was performed, showing the presence of a massive ileocolic intussusception, with increased thickness of the intestinal wall and accompanying mesentery, and enlarged lymph nodes (Figure 1A). A subsequent contrast enema, using Gastrografin® (diatrizoate meglumine and diatrizoate sodium solution) diluted with saline, was successfully performed reducing the invagination (Figure 1B). No complications occurred during the procedure.

No history or signs of SARS-CoV-2 infection was documented in the patient and in close relatives, but, due to the pandemic period, the patient and her mother were tested for SARS-CoV-2 with polymerase chain reaction (PCR) molecular testing on nasopharyngeal aspirate and swab, respectively. The mother resulted positive for SARS-CoV-2, while the patient resulted negative. A subsequent nasopharyngeal aspirate performed 24 h after the first test was performed and it confirmed the negativity for SARS-CoV-2. The patient, admitted to the ward, underwent intravenous antibiotic treatment with ceftriaxone (100 mg/kg/day) and metronidazole (20 mg/kg/day in 3 doses) until discharge.

**Figure 2:**
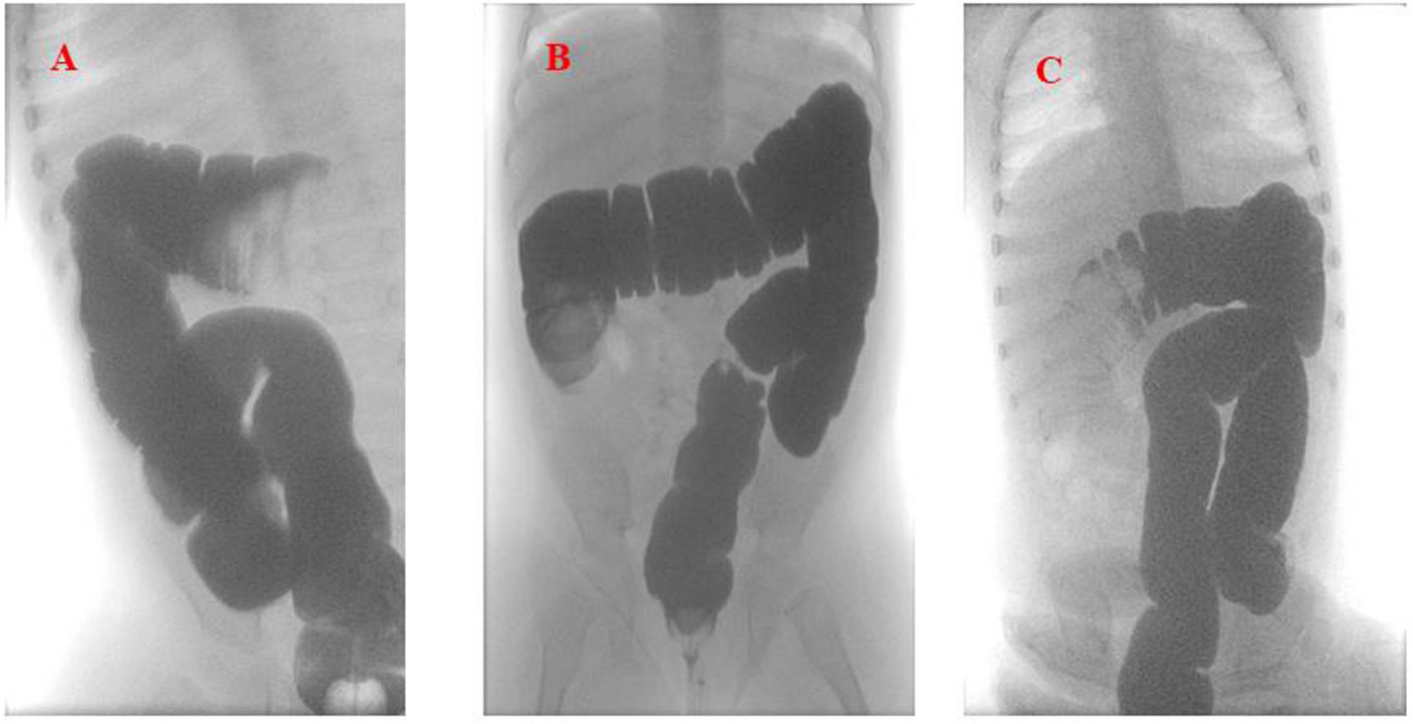
Hydrostatic reduction of intussusception using Gastrografin® contrast solution **(A)**. The first imaging shows the recurrence of the intussusception in the splenic flexure. **(B,C)** Show the relapses of the intussusception in the ascending and then back in the transverse colon, respectively.

**Figure 3:**
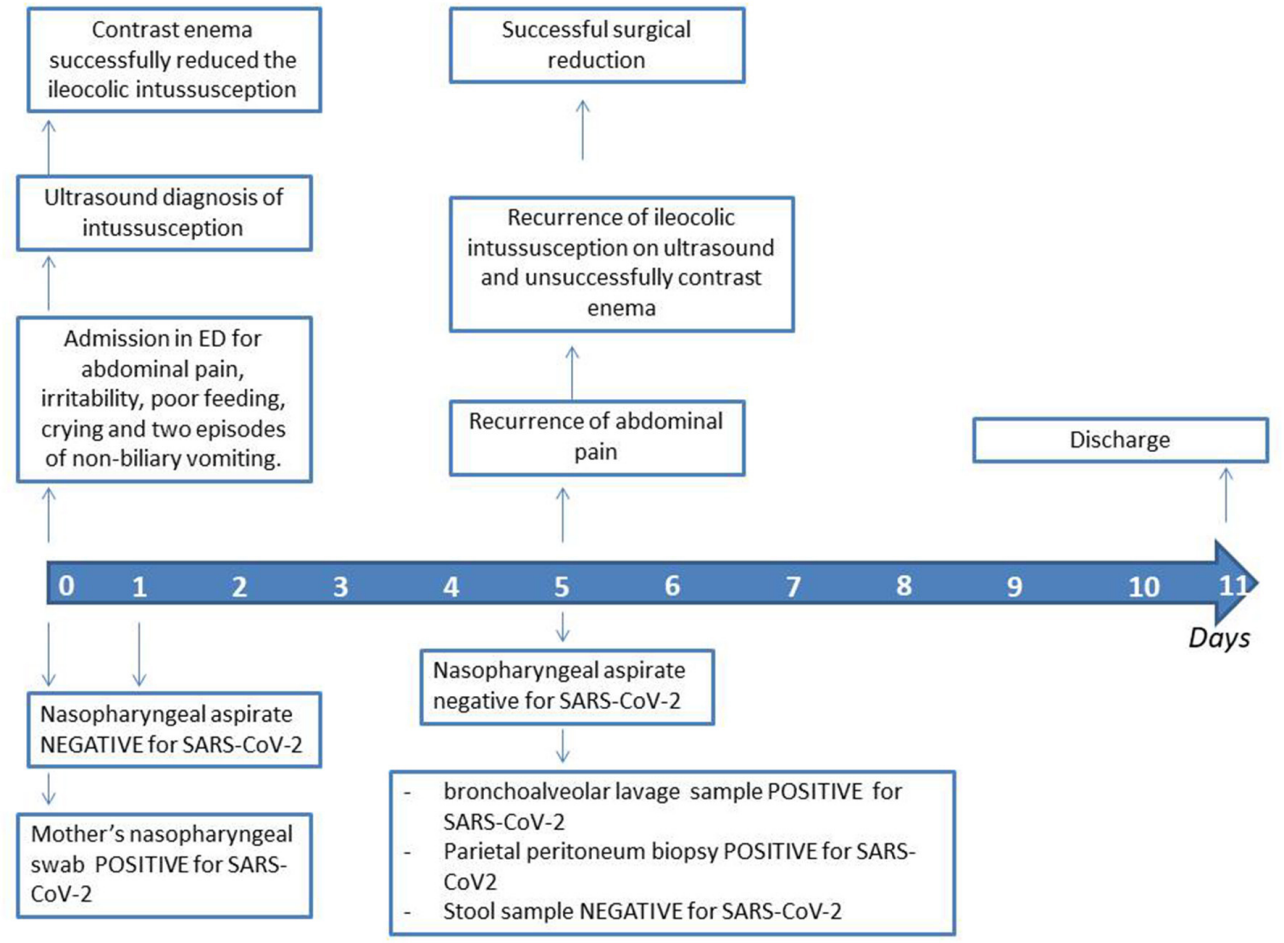
Timeline course of the patient.

An intravenous fluid replacement was administered for 2 days and then progressively reduced and interrupted on day 4 following adequate oral intake and tolerance. During the first 4 days of hospitalization, the patient experienced recurrent episodes of spiking colicky pain with spontaneous resolution in few minutes, and on day 5 an abdominal ultrasound evidenced a recurrence of the ileocolic intussusception at the level of the splenic flexure. A new non-surgical hydrostatic reduction procedure was attempted but several relapses occurred during this procedure (Figure 2). A surgical reduction was therefore indicated. Before performing the procedure, a further SARS-CoV-2 PCR molecular testing on nasopharyngeal aspirate was then obtained, with negative result. Laparotomy with manual reduction of the ileocolic invagination and ileocecopexy was subsequently performed. Macroscopically, numerous enlarged abdominal lymph nodes, especially among celiac and mesenteric ones, near the ileocecal junction, were found. Due to the COVID-19 exposure, and the persistent negativity of SARS-CoV-2 PCR molecular testing on nasopharyngeal aspirate, a molecular test was performed on a bronchoalveolar lavage (BAL) sample, a parietal peritoneum biopsy and a stool sample obtained during surgery, respectively. Positivity for SARS-CoV-2 was detected on BAL and biopsy, while stools were negative. The postoperative course was uneventful and the patient was discharged after a total of 11 days of hospitalization. A timeline of clinical course, laboratory test for SARS-CoV-2, radiological investigations and treatment of intussusception is summarized in Figure 3. During the hospital course, the patient did not develop any respiratory symptoms or signs of SARS-CoV-2 infection other than the intussusception. No further nasopharyngeal aspirate was performed for SARS-CoV-2 as the patient was completely asymptomatic.

## Discussion

Since the emergence of COVID-19 pandemic, it has been reported that children, and particularly infants, may have an asymptomatic or mild clinical presentation of the infection. Gastrointestinal symptoms, including nausea, vomiting, diarrhea, and abdominal pain have been frequently reported in the pediatric age (4). The angiotensin-converting enzyme 2 receptor (ACE-2) and transmembrane protease, serine 2 (TMPRSS2), co-expression in the same cell are essential for the entry of the virus (5). These two proteins can be expressed at a high level in the glandular cells of the gastric, duodenal, and rectal epithelium, and the enterocytes of the ileum and colon (6), so giving biologic plausibility to the gastrointestinal involvement in SARS-CoV-2 infection.

Intussusception is the leading cause of gastrointestinal obstruction in infancy. Once diagnosed, a prompt reduction of the intussusception should be performed due to the possible risk of bowel ischemia and necrosis, bowel perforation and peritonitis (7). A non-surgical reduction through pneumatic or hydrostatic enema is suggested as first line approach in the pediatric age, with a success rate close to 90% (8). Nearly 90% of intussusceptions are considered idiopathic, mainly linked to findings of lymphoid hyperplasia of the small intestine directly promoting the invagination (1). However, there is a very strong casual association between viruses, mesenteric lymphoid hyperplasia and intussusception (9). In particular, adenovirus, enterovirus, norovirus, rotavirus and human herpes virus 6 have been associated with intussusception in infants and young children (10). To the best of our knowledge, eight cases of intussusception being the presentation of COVID-19 in infants have been reported so far (11–17). The main findings from these reports are summarized in Supplementary Table 1.

The age of presentation ranges from 3 to 10 months with a male prevalence (five out of eight infants). Most reports describe an irritable baby with abdominal pain and jelly-like stools. Fever was documented in 5 cases (11, 12, 14, 15). A concurrent or a recent history of respiratory tract infection was described in only three cases (11–13). Six of the eight patients were diagnosed as ileocolic intussusception, one as ileocecal intussusception and in the last case the kind of invagination was not clarified. Surgical procedure was performed in four cases, two of which preceded by an unsuccessful pneumatic reduction (12, 15, 17). Only one case reported the patient's death due to the development of a multi-organ dysfunction syndrome (15), whereas the remaining cases were all discharged without sequelae (11–14, 16, 17).

For the direct identification of SARS-CoV-2, the PCR molecular testing should be performed on specimens taken from the upper (nasopharyngeal swab or aspirate and oropharyngeal swab) and the lower respiratory tract (BAL, tracheal aspirate and sputum) (18, 19). Since higher viral loads are usually detected in the nose compared to the throat, the World Health Organization recommends to prioritize nasopharyngeal swab over oropharyngeal swab (19, 20). All the described infants had a diagnosis of SARS-CoV-2 infection through a PCR molecular testing but only few authors reported the type of sample used for the test, in particular whether nasal or throat swabs (13, 15, 17). Furthermore, the majority of the cases (5) had no previous direct exposure to COVID-19 (12, 13, 15, 16).

Accordingly, the hereby reported 10-month old patient presented with an ileocolic intussusception, confirmed at abdomen ultrasound, with the classical presentation of abdominal pain, vomiting, lack of appetite and blood in stools. Of note, she was apyretic, and neither previous or current respiratory symptoms, nor direct exposure to COVID-19 infected patients were reported in either the patient and her close relatives. Following our Institutional norms, a PCR testing was performed on nasopharyngeal aspirate and swab from the patient and her mother, respectively, and only the mother resulted positive for SARS-CoV-2. In occasion of the surgical manual reduction following the relapse of symptoms, a PCR molecular testing on BAL and biopsy specimens were performed and both resulted positive for SARS-CoV-2. To the best of our knowledge, this is the first report in which SARS-CoV-2 was detected in these specimens in infants with intussusceptions. Accordingly, we may suggest to expand the diagnostic workout in cases where the nasal swab of the patient with intestinal intussusception is negative, but there is strong evidence of recent direct exposure to SARS-CoV-2, up to specific gut bioptic specimen, when feasible due to intercurring surgical procedures, as in our case. Without the clear positivity to SARS-CoV-2 observed in the mother, no further tests would have probably been conducted on this patient, with a possible underestimation of the diagnosis. Almost unexpectedly, the stools specimens of our patient resulted negative for SARS-CoV-2 even if 8 out of 10 COVID-19 positive children have been reported with positive rectal swabs, in spite of negative nasopharyngeal swabs (21). Accordingly, although viral shedding in COVID-19 patients primarily occurs through nasal secretions, a feco-oral route of transmission has also been postulated.

The mechanism linking the development of intussusception in pediatric patients with SARS-COV-2 infection is not fully understood (16). Previous reports have suggested an association between viral infections, whichever the nature, and a delay in gut peristaltic movements, finally favoring a spontaneous gliding of adjacent bowel loops toward each other (22).

## Conclusion

As described, COVID-19 may be implicated in the development of intussusception in infants, even in absence of a systemic and respiratory involvement. Due to the possible link with infectious pathogens, testing for viral pathogens, including SARS-CoV-2, should be performed in infants with symptoms consistent with intussusception. In those infants with a history of direct SARS-CoV-2 exposure, we hereby suggest to enlarge the diagnostic workout for SARS-CoV-2 to feasible samples in case nasopharyngeal swab or aspirate result negative, in order to achieve a precise diagnosis, whether positive or negative.

## Data Availability Statement

The original contributions presented in the study are included in the article/Supplementary Materials, further inquiries can be directed to the corresponding author.

## Ethics Statement

Ethical approval was not provided for this study on human participants because it is a case report. Written informed consent to participate in this study was provided by the participants' legal guardian/next of kin.

## Author Contributions

MC, ACo, and LC designed the topic and wrote the first draft of the manuscript. MC, PM, and CA contributed to the clinical diagnosis. AM and EL contributed to patient management. ACa performed the radiological studies. PM, CA, AM, and EL revised the manuscript. All authors read and approved the final manuscript and agree to be accountable for the content of the work.

## Conflict of Interest

The authors declare that the research was conducted in the absence of any commercial or financial relationships that could be construed as a potential conflict of interest.

## Publisher's Note

All claims expressed in this article are solely those of the authors and do not necessarily represent those of their affiliated organizations, or those of the publisher, the editors and the reviewers. Any product that may be evaluated in this article, or claim that may be made by its manufacturer, is not guaranteed or endorsed by the publisher.
