# Supplementary material for: Intussusception in an Infant With SARS-CoV-2 Infection: A Case Report and a Review of the Literature
Source: Front Pediatr. 2021 Aug 4;9:693348. doi: 10.3389/fped.2021.693348 (PMC8371323; doi:10.3389/fped.2021.693348)
Supplement: Supplementary file 2 [file Table_1.DOCX]

**Table 1.** Summary of the reported cases of intussusception in infants and SARS-CoV-2 infection.

| **Author, nation, reference** | **Number of patients** | **Sex and age** | **Clinical presentation** | **Type of intussusception, diagnosis** | **SARS-CoV 2 diagnosis** | **Prior SARS-CoV 2 contact** | **Treatment** | **Outcome** |
| --- | --- | --- | --- | --- | --- | --- | --- | --- |
| Bazuaye-Ekwuyasi EA, USA, (11) | 1 | M, 9-month-old | Congestion, cough, and sneezing for 4 days and fever. Vomit, episodic abdominal pain, decreased oral intake, and blood streaked stool. | Ileocolic intussusception, abdominal radiograph and abdominal ultrasound | Polymerase chain reaction (PCR) molecular testing  (not clarified the type of sample) | Yes (from relatives) | Hydrostatic Reduction | Discharged |
| Mercado-Martínez I, Mexico, (12) | 2 | M, 8-month-old  F, 7-month- old | Feverish feeling starting 2 days prior to admission, non-biliary episodes of vomiting, stools with currant jelly appearance.  1 week prior upper respiratory tract infection  Intermittent crying, stools with currant jelly appearance, fever and non-biliary vomiting.  No history of respiratory infection. | Ileocolic intussusception, abdominal ultrasound  Ileocolic intussusception, abdominal radiograph | PCR molecular testing  (not clarified the type of sample)  PCR molecular testing  (not clarified the type of sample) | None  None | Surgical treatment  Surgical treatment | Discharged  Discharged |
| Moazzam Z, Pakistan, (13) | 1 | M, 4-month-old | Acute onset, intermittent, severe, crampy abdominal pain, inconsolable crying. Poor feeding. Bloody stools.  1 week prior upper respiratory tract infection | Ileocolic intussusception, abdominal ultrasound | PCR molecular testing on nasal swab | None | Pneumatic reduction (2 procedures) | Discharged |
| Athamnah MN, Jordan, (14) | 1 | M, 3-month-old | Vomiting, abdominal distension, fever, abdominal pain.  No history of respiratory infection. | Ileocolic intussusception, abdominal radiograph and abdominal ultrasound | PCR molecular testing  (not clarified the type of sample) | Yes (her mother had a flu-like syndrome 10 days before) | Pneumatic reduction | Discharged |
| Cai X, China, (15) | 1 | F, 10-month-old | Paroxysmal crying and restlessness, vomiting, and currant jelly-like stool. Fever.  No history of respiratory infection | Ileocolic  Intussusception, abdominal ultrasound | PCR molecular testing on throat swabs. | None | Pneumatic reduction then surgery | Died because of the development multiorgan dysfunction syndrome |
| Martínez-Castaño I, Spain, (16) | 1 | M, 6-month-old | Abdominal cramps, currant jelly stool, vomiting.  No history of respiratory infection | Ileocecal intussusception,  abdominal ultrasound | PCR molecular testing  (not clarified the type of sample) | None | Hydrostatic reduction | Discharged |
| Makrinioti H, United Kingdom, (17) | 2 (but 1 is the same described by Cai et al.) | F, 10-month-old | Lethargy, poor peripheral perfusion, unresponsive to pain. Bilious vomiting and red currant jelly stool.  Recent history of intermittent coryzal symptoms and bilateral conjunctivitis. | Abdominal ultrasound | PCR molecular testing on nasal and throat swabs | Yes (mother, siblings with URTI and fever 3 weeks before presentation) | Pneumatic reduction (failed)  Surgical reduction: a malrotation was also  identified for which a Ladd’s procedure was performed. | Discharged |
